# Transplantation of Stem Cell Spheroid-Laden 3-Dimensional Patches with Bioadhesives for the Treatment of Myocardial Infarction

**DOI:** 10.34133/bmr.0007

**Published:** 2024-03-04

**Authors:** Hye Ran Jeon, Jeon Il Kang, Suk Ho Bhang, Kyung Min Park, Dong-Ik Kim

**Affiliations:** ^1^ Department of Health Sciences and Technology, Samsung Advanced Institute for Health Sciences and Technology (SAIHST), Sungkyunkwan University, Seoul 06355, Republic of Korea.; ^2^ Department of Bioengineering and Nano-Bioengineering, College of Life Sciences and Bioengineering, Incheon National University, 119 Academy-ro, Yeonsu-gu, Incheon 22012, Republic of Korea.; ^3^ School of Chemical Engineering, Sungkyunkwan University, Suwon 16419, Republic of Korea.; ^4^ Research Center for Bio Materials & Process Development, Incheon National University, 119 Academy-ro, Yeonsu-gu, Incheon 22012, Republic of Korea.; ^5^ Division of Vascular Surgery, Sungkyunkwan University School of Medicine, Samsung Medical Center, Seoul 06351, Republic of Korea.

## Abstract

Myocardial infarction (MI) is treated with stem cell transplantation using various biomaterials and methods, such as stem cell/spheroid injections, cell sheets, and cardiac patches. However, current treatment methods have some limitations, including low stem cell engraftment and poor therapeutic effects. Furthermore, these methods cause secondary damage to heart due to injection and suturing to immobilize them in the heart, inducing side effects. In this study, we developed stem cell spheroid-laden 3-dimensional (3D) patches (S_3DP) with biosealant to treat MI. This 3D patch has dual modules, such as open pockets to directly deliver the spheroids with their paracrine effects and closed pockets to improve the engraft rate by protecting the spheroid from harsh microenvironments. The spheroids formed within S_3DP showed increased viability and expression of angiogenic factors compared to 2-dimensional cultured cells. We also fabricated gelatin-based tissue adhesive biosealants via a thiol-ene reaction and disulfide bond formation. This biosealant showed stronger tissue adhesiveness than commercial fibrin glue. Furthermore, we successfully applied S_3DP using a biosealant in a rat MI model without suturing in vivo, thereby improving cardiac function and reducing heart fibrosis. In summary, S_3DP and biosealant have excellent potential as advanced stem cell therapies with a sutureless approach to MI treatment.

## Introduction

Cardiovascular diseases, responsible for 17.7 million deaths annually, have emerged as the primary global cause of mortality. This number is expected to increase to 23.6 million by 2030, surpassing that of cancer and other ailments [1]. Among cardiovascular diseases, myocardial infarction (MI; abbreviations are listed in Table S1) is a representative cardiac ischemic disease caused by coronary artery occlusion [2–5]. MI induces a lack of oxygen and nutrient supply, causing myocardial necrosis, loss of cardiac function, and eventual heart failure [2,3]. Furthermore, unlike other organs, the regenerative ability of the damaged heart is limited because adult cardiomyocytes have low proliferative and self-renewal capacities [3,5–8]. Conventionally, MI is treated using a combination of pharmacological and surgical interventional therapies, such as balloon angioplasty, coronary bypass, stent insertion, and heart transplantation, to enhance patient prognosis [2,8–10]. However, these therapies have not been able to be a fundamental treatment to regenerate injured myocardium with its function, and postoperative management is important to reduce infarct size [4]. Therefore, it is necessary to develop treatments that restore the underlying damaged tissue and its function.

Over the last few decades, stem cell transplantation has been highlighted as an alternative source of the myocardium to treat ischemic hearts because of their pluri- or multi-potency [8,9]. Mesenchymal stem cells (MSCs) have been extensively used as promising therapeutic agents for various diseases, extending beyond MI [9]. Although stem cells have been transplanted to treat MI, the low retention of stem cell delivery (5% to 10%) in the myocardium limits their applications [4–6]. Therefore, various technologies, including spheroid formations, injectable biomaterials, cell sheets, and cardiac patches, have emerged as more effective approaches to deliver stem cells with improved retention rates to promote the restoration of infarcted myocardial functions [4,11–14].

Stem cell spheroids are dense 3-dimensional (3D) cell clusters formed by cell-to-cell aggregation. Fabricating spheroids promotes stem cell survival and retention, and physiological and metabolic functions compared to single stem cells [15,16]. Injectable biomaterials, such as polymeric hydrogels, have attracted considerable attention as delivery carriers for cells and spheroids. They can promote retention rates and biological functions by providing a 3D artificial extracellular matrix (ECM) that can recapitulate cell-to-matrix interactions and physically support cells and spheroids [1,16,17]. However, these methods inherently require syringe injection, which causes shear stress on the cells and spheroids, leading to structural damage to the spheroid and reduced cell viability [17,18]. Furthermore, physical injury caused by the injection can cause secondary damage, bleeding, and myocardial perforation, leading to possible adverse cardiac events [7,19]. As with other strategies, cell sheets and cardiac patches have been widely utilized due to their improved cell-to-cell communication and more powerful paracrine effects, improving prognosis compared to stem cell solution therapy [9,13]. Although these strategies can directly or indirectly deliver stem cells and their cytokines/growth factors to treat MI, suturing is essential to immobilize them onto the epicardial surface and induce secondary damage [9,20,21]. Therefore, it is necessary to develop advanced stem cell therapies to improve the stem cell engraftment rate and paracrine effects using a sutureless approach.

Polymeric hydrogel-based biosealants have recently emerged as a promising alternative for suturing and stapling because of their sol-gel phase transition, bioactivity, and strong tissue adhesion [22,23]. Therefore, they have been used to prevent gas/liquid leakage and bleeding and immobilize implantable medical devices on target tissues [24]. Currently, various tissue adhesive biosealants, such as Tisseel, Coseal, Duraseal, Progel, and others, have been successfully approved by the Food and Drug Administration and are widely used in clinics [24]. However, these commercialized biosealants have relatively lower tissue adhesiveness when applied to myocardial tissue owing to the presence of the body or pericardial fluid and the dynamic pulsation of the heart [20,25–27]. Therefore, there is a need to develop advanced functional biosealants with strong tissue adhesion. Toward this, the biosealant that will be applicated for heart tissue has to meet some requirements, such as (a) rapid and easy application for clinical compliance, (b) biocompatibility of polymer and chemistry, (c) mechanical similarity to surrounding tissue and elasticity to heartbeats, (d) strong tissue adhesion on the heart surface under the pericardial fluid and the dynamic heartbeats, and (e) biodegradability.

**Fig. 1.**
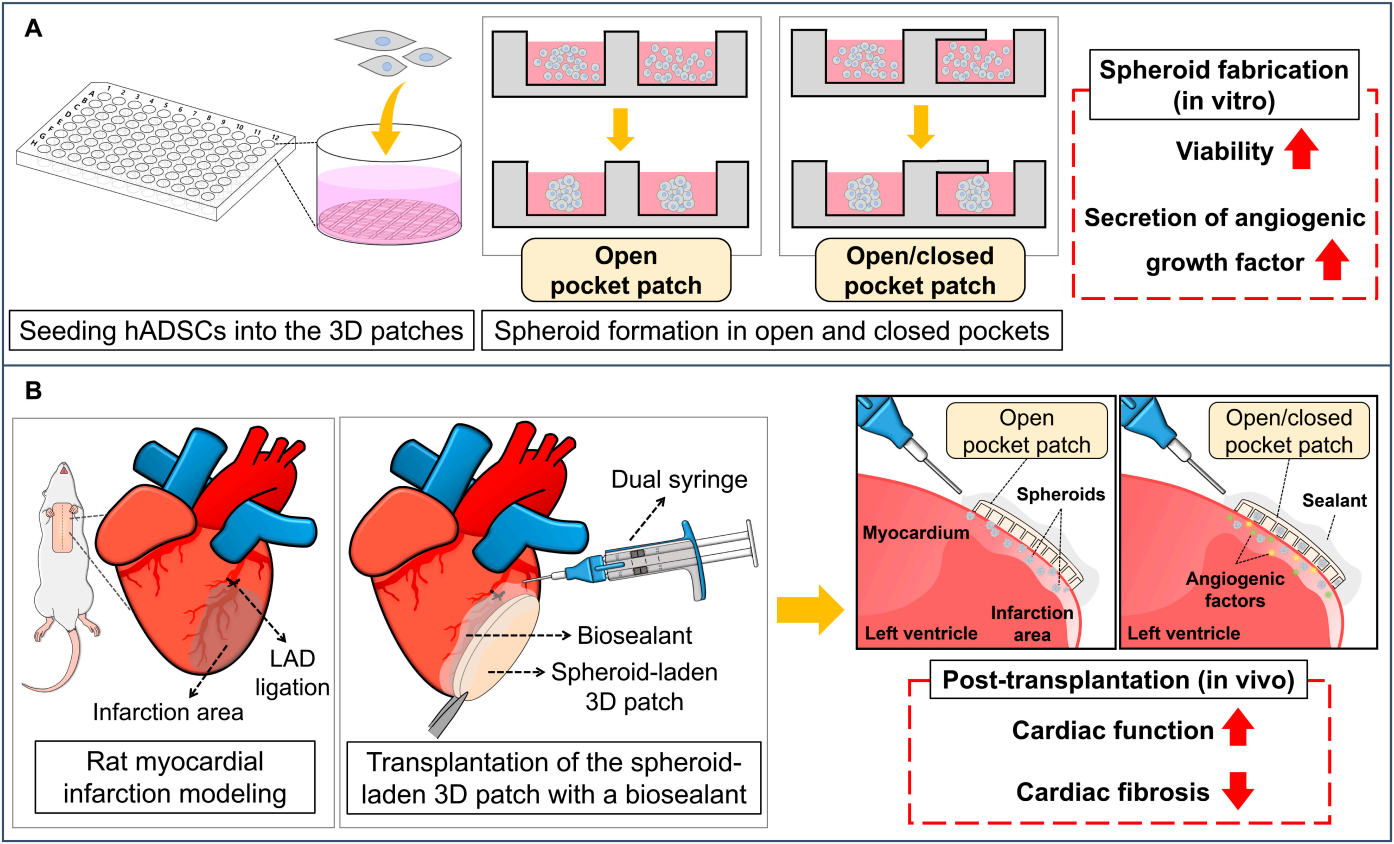
Schematic illustration of open pocket and open/closed pocket 3D patches laden with human adipose-derived stem cell (hADSC) spheroids for effective MI treatment. (A) By forming hADSC spheroids in the open or closed pocket of the 3D patches, cell viability and expression of the angiogenic growth factors were increased. (B) The transplantation of S_3DP using a biosealant onto the infarcted region of the rat heart and its therapeutic effects.

In this study, we developed 2 types of stem cell spheroid-laden 3D patches (S_3DP): (a) open pockets to directly deliver stem cell spheroids with an increased paracrine effect, and (b) open/closed pockets to enhance the engraft rates of spheroids by protecting them from harsh microenvironments (Fig. 1A). The stem cell spheroids formed within these patches revealed high cell viability with improved bioactivities, such as the expression of genes and proteins and paracrine ability related to angiogenesis, in vitro. We also developed a tissue adhesive biosealant as a sutureless technique to immobilize functional S_3DP on the heart tissue without physical damage. Finally, we transplanted S_3DP into a rat MI model using a biosealant and demonstrated its effect on cardiac function and fibrosis in vivo (Fig. 1B). The entire S_3DP preparation and transplantation process for MI treatment is depicted in Fig. 1.

**Table 1.** Sample codes and experimental condition of S_3DP

Sample codes	Pocket type	Patch swelling time	Cell number (cells/patch)	Incubation time
2D	-	At least 5 min	3 × 10^5^	24 h
S_OP	Open			
S_OPCL	Open/closed			

**Table 2.** Sample codes and compositions

Sample codes	MI induction	Treatment of biosealant	3D patch transplantation (pocket type)	Spheroid
Normal	X	X	X	X
MI	O	X	X	X
SO	O	O	X	X
PO	O	O	O (Open/closed pocket)	X
S_OP	O	O	O (Open pocket)	O
S_OPCL	O	O	O (Open/closed pocket)	O

## Materials and Methods

### Materials

For fabrication of the patch and stem cell spheroid, Dulbecco’s modified Eagle’s medium (DMEM), fetal bovine serum (FBS), penicillin/streptomycin antibiotics (P/S), and trypsin were purchased from Gibco BRL (Gaithersburg, MD, USA). Phosphate-buffered saline (PBS) was purchased from Biosesang (Seongnam, Korea). Human adipose-derived stem cell (hADSC) was obtained from Lonza (Walkersville, MD, USA). The biopsy punch was purchased from Kai Medical (Gifu, Japan). For the in vitro spheroid study, TRIzol was purchased from Ambion (Austin, TX, USA). Chloroform, isopropyl alcohol, ethanol, and β-mercaptoethanol were purchased from Sigma-Aldrich (St. Louis, MO, USA). AccuPower RocketScript Cycle RT PreMix and AccuPower 2X Greenstar qPCR MasterMix were purchased from Bioneer (Daejeon, Korea). PRO-PREP Protein Extraction Solution was obtained from iNtRON Biotechnology (Seongnam, Korea). Bradford reagent and 4× Laemmli sample buffer were obtained from Bio-Rad (Hercules, CA, USA). Five percent skim milk was purchased from BD Difco (Detroit, MD, USA). Antibodies of glyceraldehyde 3-phosphate dehydrogenase (GAPDH), Bcl-2-associated X (BAX), and hepatocyte growth factor (HGF) were purchased from Abcam (Cambridge, MA, USA). Antibodies against E-cadherin and B-cell lymphoma 2 (BCL-2) were purchased from Cell Signaling Technology (Danvers, MA, USA), and the vascular endothelial growth factor (VEGF) antibody was purchased from Santa Cruz Biotechnology (Dallas, TX, USA). Goat anti-mouse IgG-HRP and goat anti-rabbit IgG-HRP antibodies were purchased from Bethyl Laboratories (Montgomery, TX, USA). The ECL reagent WESTSAVE UP was purchased from AbFrontier (Seoul, Korea). The x-ray films were obtained from AGFA HealthCare NV (Mortsel, Belgium). The Proteome Profiler Human Angiogenesis Array Kit was purchased from R&D Systems (Minneapolis, MN, USA). For in vivo animal study, 8- to 10-week-old male Sprague–Dawley rats were supplied by Orient Bio Inc. (Seongnam, Korea). 6-0 polypropylene, 4-0 surgifit, and 6-0 black silk sutures were obtained from Ailee (Busan, Korea). Ten percent formalin and paraffin were purchased from Sigma-Aldrich (St. Louis, MO, USA). Xylene was obtained from Daejung (Siheung, Korea). The hematoxylin and eosin (H&E) staining kit was purchased from Abcam (Cambridge, MA, USA), and Masson’s trichrome was purchased from Dako (Hamburg, Germany). A Millipore Milli-Q purification system treated with water was used for all the experiments.

For polymer synthesis and characterization, gelatin (Gtn, type A from porcine skin, <300 bloom), cystamine dihydrochloride (CYS), 6-maleimidohexanoic acid (MHA), 1-ethyl-3-(3-dimethylaminopropyl)carbodiimide hydrochloride (EDC), N-hydroxysuccinimide (NHS), DL-dithiothreitol (DTT), and deuterium oxide (D_2_O) were purchased from Sigma-Aldrich (St. Louis, MO, USA). Dulbecco’s phosphate-buffered saline (DPBS) was obtained from Gibco (Grand Island, NY, USA). Dialysis membranes (molecular cutoff = 3.5 kDa) were supplied by Spectrum Laboratories (Rancho Dominguez, CA, USA). Hydrochloric acid (HCl, 1N) was obtained from Daejung (Siheung, Korea). 5,5′-Dithio-bis(2-nitrobenzoic acid) (Ellman’s reagent) was provided by Thermo Fisher Scientific (Rockford, IL, USA). For the in vitro cell study, human dermal fibroblast (HDF) was purchased from Lonza (Walkersville, MD, USA). DMEM, newborn calf serum (NBCS), P/S, and 0.25% trypsin-EDTA were supplied by Gibco (Grand Island, NY, USA). The Live/Dead kit was obtained from Invitrogen (Grand Island, NY, USA). The WST-1 cell proliferation kit was purchased from Roche (Basel, Switzerland). For the in vivo animal study, 5-week-old female C57BL mice were obtained from Koatech (Gyeonggi-do, Korea). Isoflurane (Forane Solution) was purchased from Hana Pharm Co. (Seongnam, Korea). Formalin (10%) was purchased from Sigma-Aldrich (St. Louis, MO, USA). Hematoxylin H and aqueous eosin Y solutions were purchased from Sigma-Aldrich (St. Louis, MO, USA).

### Fabrication of open and open/closed pocket patches for stem cell spheroid encapsulation

In our previous study, we developed an elastomeric bioscaffold (TPU-CEC363) with tunable biodegradability and elasticity for noninvasive stem cell-based therapy [28]. In this study, developed elastomers were used as bioinks to fabricate stem cell spheroid-encapsulating patches. Briefly, we synthesized TPU-CEC363 using poly(ethylene glycol) (PEG; Sigma-Aldrich), ε-caprolactone (Tokyo Chemical Industry, Tokyo, Japan), iron (III) acetylacetonate (Sigma-Aldrich), and toluene (Daejung). TPU-CEC363 was then synthesized using the tri-block copolymer, toluene, and hexamethylene diisocyanate. As a result, TPU-CEC363 containing 50% each of PEG and polycaprolactone was prepared, and the synthesized TPU-CEC363 was extruded to a diameter of 1.75 mm [28]. To fabricate the 3D patches, we set the printing program to continuously eject the bioink under the following nozzle conditions. We used a printing nozzle with a diameter of 300 μm to print the patches (25 × 25 mm) with a thickness of 100 μm in each layer. The 3D patches were fabricated using FDM Vis Power Plus (Vision Technology Korea, Daejeon, Korea) with a flow rate of 1.196 and a nozzle temperature of 230 °C.

### Characterization of open and open/closed pocket patches

The 3D-printed patches with a size of 25 × 25 mm were supplied by the Korea Institute of Science and Technology and Vision Technology Korea. The 3D patches were cut using a biopsy punch with a diameter of 4 mm, and the number of pockets in each patch was observed under a light microscope (CKX41, Olympus, Tokyo, Japan) at ×40 magnification. The swelling ability was performed with 1× PBS, and photographs of the pockets were captured using a light microscope (CKX41, Olympus) at ×100 magnification and Infinity analyze software (Lumenera Corporation, Ottawa, Canada). The pocket size was analyzed using ImageJ software (National Institutes of Health, Bethesda, MD, USA).

### Preparation of 3D patch and spheroid formation in 3D patch

The 3D patches with a diameter of 4 mm were placed into each well of a 96-well plate, and the 96-well plate without a lid was placed in a sterilized pack and sterilized with ethylene oxide gas at 37 °C for 3 h. A 96-well plate containing sterilized 3D patches was refrigerated and protected from light to slow degradation. Before use, the 3D patches were swollen by adding 250 μl of 1× PBS to the wells of a 96-well plate. The 3D patches in PBS were centrifuged at 1,500 rpm for 5 min to remove air within the pockets, and the PBS was replaced with 200 μl of serum-free DMEM. The hADSCs were cultured with DMEM containing 10% (v/v) FBS and 1% (v/v) P/S in a 5% carbon dioxide (CO_2_) incubator at 37 °C. The medium was changed every 2 days, and hADSCs from passages 4 to 7 were used in the experiments. Serum-free DMEM (50 μl) containing 3 × 10^5^ cells was dispensed over the swollen 3D patches within the wells of a 96-well plate. The hADSCs entered the pockets of the 3D patches after centrifugation at 1,500 rpm for 5 min, and spheroids were formed in the open and open/closed pockets after 24 h. The sample codes and experimental conditions are described in Table 1.

### Characterization of stem cell spheroid

We observed the spheroid morphology under a light microscope (CKX41, Olympus) at ×40 magnification after 24 h. Images were captured to confirm the diameter of the spheroids using a light microscope (CKX41, Olympus) at ×100 magnification and Infinity analyze software (Lumenera Corporation), and the diameter was analyzed using ImageJ software.

### Quantitative reverse transcription-polymerase chain reaction

Total RNA was extracted using 1 ml of TRIzol and 200 μl of chloroform. After centrifugation at 11,000 rpm for 10 min at 4 °C, 80% (v/v) isopropyl alcohol (in water) and 75% (v/v) ethanol (in water) were used for washing. The samples were then dissolved in RNase-free water. Total RNA (1 μg) and the AccuPower RocketScript Cycle RT PreMix were used to synthesize complementary DNA. The AccuPower 2X Greenstar qPCR MasterMix and QuantStudio 6 Flex Real-Time PCR System (Applied Biosystems, Waltham, MA, USA) were used for quantitative reverse transcription-polymerase chain reaction (qRT-PCR). The relative gene expression was analyzed using the 2^−ΔΔCT^ method, and GAPDH served as the internal control. The primer sequences are described in Table S2.

### Western blot analysis

Spheroids were extracted using PRO-PREP Protein Extraction Solution to prepare protein samples. Protein concentration was determined using the Bradford assay. Protein samples were boiled at 100 °C for 10 min in 4× Laemmli sample buffer containing β-mercaptoethanol, and samples were loaded onto a sodium dodecyl sulfate-polyacrylamide gel electrophoresis (SDS-PAGE) gel. After gel electrophoresis, proteins were transferred from gel to nitrocellulose membrane, and membranes were continuously blocked with 1× TBS-T containing 5% skim milk for 1 h at room temperature (RT). The membranes were incubated overnight at 4 °C with primary antibodies: anti-GAPDH (1:3,000), anti-E-cadherin (1:500), anti-BAX (1:250), anti-BCL-2 (1:250), anti-HGF (1:500), and anti-VEGF (1:250). The membranes were washed with 1× TBS-T for 45 min and incubated for 1 h at RT with goat anti-mouse IgG-HRP (1:5,000) and goat anti-rabbit IgG-HRP (1:10,000) secondary antibodies. After incubation, the membranes were washed with 1× TBS-T for 45 min, and protein bands were detected using the ECL reagent WESTSAVE UP and an x-ray film. To analyze protein expression, GAPDH and ImageJ software were used as a housekeeping control and an analysis tool.

### Angiogenesis antibody array

To profile angiogenesis-related proteins in the conditioned medium (CM) of the spheroids, we used the Proteome Profiler Human Angiogenesis Array. After spheroid formation in the 3D patches, the cells were carefully washed with 1× PBS, and the spheroids in the 3D patches were incubated with fresh serum-free DMEM for 24 h to harvest the CM. The protein concentration of the harvested CM was measured by Bradford assay. The array membranes were blocked according to the manufacturer’s instructions, and the buffer was aspirated. The sample/antibody mixtures were added to the membranes and incubated overnight at 4 °C. The following day, we washed each membrane with 1× wash buffer, and membranes were incubated with streptavidin-HRP for 30 min at RT. After washing, the membranes were covered with Chemi reagent mix and exposed to x-rays. Pixel densities on the developed x-ray film were quantified at each spot using ImageJ software.

### Synthesis and characterization of biosealant polymers

To fabricate the biosealant for fixing S_3DP on the heart surface, we synthesized thiolated gelatin (GtnSH) and maleimide-conjugated gelatin (GtnMI) via EDC/NHS-mediated conjugation of thiol and maleimide groups to Gtn backbone. To synthesize GtnSH, we dissolved 250 mg of gelatin in 125 ml of deionized water (DIW) at 37 °C. After Gtn dissolution, 450.5 mg of CYS (2.0 mmol) was dissolved in 2.5 ml of DIW and added into the Gtn solution. We homogenously mixed them for 10 min at 37 °C. Next, 192.0 mg of EDC (1.0 mmol) and 116.0 mg of NHS (1.0 mmol) were dissolved in DIW (2.5 ml) in a 10-ml vial, respectively. EDC and NHS solutions were sequentially added to Gtn/CYS and allowed to react for 2 h at 500 rpm. After the conjugation reaction, we injected a DTT solution (617.0 mg, 4.0 mmol) dissolved in 5 ml of DIW into the reactant and incubated it for 24 h at 500 rpm. This reactant was serially dialyzed using a dialysis membrane bag (molecular weight cutoff = 3.5 kDa) against a 5 mM HCl solution for 36 h, followed by a 1 mM HCl solution for 24 h. The solution was changed twice to remove unconjugated residual chemicals, such as unconjugated CYS, by-products of the EDC/NHS reaction, and unreacted DTT molecules. To obtain the GtnSH polymer, we froze the dialyzed GtnSH solution at −80 °C and lyophilized it for 5 to 6 days. The obtained polymers were stored in a desiccator until use.

To synthesize GtnMI, we dissolved 250 mg of Gtn and 211.2 mg of MHA (1.0 mmol) in 50 ml and 120 ml of DPBS solution at 37 °C, respectively. Each solution was mixed for 10 min at 37 °C. Then, 230.1 mg of EDC (1.2 mmol) and 161.1 mg of NHS (1.4 mmol) were dissolved in 5 ml of DPBS within a 10-ml vial, respectively. The EDC solution was mixed with the NHS solution and added to the Gtn/MHA mixture. After 2 h of reaction, the reactant was dialyzed using a dialysis membrane bag (molecular weight cutoff = 3.5 kDa) against DIW for 72 h to remove the unconjugated MHA molecules and by-products of the EDC/NHS reaction. After dialysis, the GtnMI solution was frozen at −80 °C and lyophilized for 5 to 6 days. Lyophilized GtnMI were stored in a deep freezer at −80 °C without light.

A proton nuclear magnetic resonance (^1^H-NMR) spectrometer (Agilent 400–MR, Agilent Technologies, CA) was used to characterize the chemical structures of GtnSH and GtnMI. We prepared 600 μl of GtnSH and GtnMI solutions (10 mg/ml) using D_2_O and analyzed each chemical structure.

Next, Ellman’s assay was performed to determine the functional groups of the biosealant polymers. To measure the thiol content of GtnSH, we prepared 0 to 1 mg/ml L-cysteine solutions using DIW to obtain a standard curve. Next, 100 μl of GtnSH solution (1 mg/ml) dissolved in DIW and standard solution were mixed with 100 μl of Ellman’s reagent solution. After 20 min of reaction in the dark, the absorbance of the samples was measured at 405 nm using an ultraviolet (UV) spectrophotometry (Multiskan EX, Thermo Fisher Scientific, Rockford, IL, USA). Thiol content was calculated from a thiol standard curve at known L-cysteine concentrations (0.001 to 0.04 mg/ml). To measure the maleimide content of GtnMI, we performed a modified Ellman’s assay by introducing a thiol-ene reaction. We prepared a 0.04 mg/ml L-cysteine solution as a substrate for the thiol-ene reaction. After mixing 50 μl of 0.04 mg/ml L-cysteine solution with 50 μl of standard MHA solution or 50 μl GtnMI solution, we incubated them to induce the thiol-ene reaction for 10 min without light. We next added 50 μl of Ellman’s reagent solution to quantify the degree of thiol group reduction depending on the content of the maleimide group in the standard solution and GtnMI solution. After 20 min of incubation in the dark, we measured the absorbance at 405 nm using UV spectrophotometry (Multiskan EX, Thermo Fisher Scientific, Rockford, IL, USA). The maleimide content was calculated from the maleimide standard curve at known MHA concentrations (0.005 to 0.04 mg/ml).

### Fabrication of tissue adhesive biosealant

We fabricated a tissue adhesive biosealant by simply mixing GtnSH, GtnMI, and calcium peroxide (CaO_2_) solutions (volume ratio of GtnSH:GtnMI:CaO_2_ = 10:9:1). In this system, CaO_2_ was used as an additional crosslinker to enhance the mechanical property of the biosealant. We first dissolved 20 mg of GtnSH and GtnMI in 200 μl and 180 μl of prewarmed DPBS, respectively. We loaded the GtnSH solution into a 1-ml commercial syringe (Korea Vaccine Co., Ltd., Gyeonggi-do, Korea). Next, a 20-μl CaO_2_ solution (0 to 2 wt%) prepared using 1 M Tris-HCl was mixed with 180 μl of GtnMI solution and loaded into another 1-ml commercial syringe. Finally, both syringes were equipped with a dual syringe kit and the solution was injected to fabricate the tissue adhesive biosealant.

### Rheological analysis of biosealant

We determined the elastic modulus (*G*′) of the biosealant using a rheometric fluid spectrometer (DHR-1, TA instruments, New Castle, DE, USA) in oscillatory mode. We prepared each 150-μl GtnSH and GtnMI/CaO_2_ solution and equipped them into a dual syringe kit. Next, we injected a biosealant solution on the parallel plate (diameter, 20 mm) with a gap of 600 μm and performed dynamic time sweeps on samples depending on CaO_2_ concentrations at a frequency of 0.1 Hz and a strain of 0.1% at 37 °C. To prevent solvent evaporation, a solvent trap was filled with DIW.

### Tissue adhesive test of biosealant

The tissue adhesiveness of the biosealant was measured using a universal testing machine (UTM, UNITEST M1, TEST ONE, Busan, Korea) with a load cell sensor (LCK1205-K010) according to the modified ASTM standard F2255-05 method. We prepared the decellularized porcine skin (round shape, 10 mm diameter) using a 0.1% SDS solution and a freeze dryer. Next, the decellularized porcine skin was attached to an acrylic plate (1 × 4 cm) using cyanoacrylate. After swelling of decellularized porcine skin, we injected a biosealant solution using a dual syringe filled with 100 μl of GtnSH and GtnMI/CaO_2_ solutions between 2 pieces of decellularized porcine skin. We stabilized samples for 30 min under a force of 100 g at 37 °C within a humid chamber. The tissue adhesion force was measured at a speed of 1 mm/min. To evaluate the feasibility of the tissue adhesive biosealant in vivo, we treated biosealant to various mouse tissues, such as the heart, liver, spleen, lung, kidney, and skin. Various organs and skin tissues were harvested and rinsed with DPBS to remove blood and body fluids. Next, we cut the tissues in half and treated the tissue surfaces or cut edges with biosealants. We stabilized them for 5 min at 37 °C within an incubator and lifted them to confirm the tissue adhesion of the biosealant. The animal study was performed according to a protocol approved by the Institutional Animal Care and Use Committee (IACUC) of the Incheon National University (INU-ANIM-2022-02).

### Cytocompatibility of biosealant

We evaluated the cytocompatibility of polymers and biosealants using HDFs. All reagents and solutions used in this experiment were sterilized by UV irradiation for 15 min or syringe filtration with a pore size of 0.2 μm. To assess the cytotoxicity of GtnSH and GtnMI, the WST-1 assay (Roche) was performed according to the manufacturer’s instructions and our previous reports [29,30]. Briefly, we seeded 1.0 × 10^4^ cells in a 96-well plate (SPL Life Sciences, Korea) with 200 μl of DMEM-HG supplemented with 10% (v/v) NBCS and 1% (v/v) P/S at 37 °C in 5% CO_2_. We prepared different concentrations (0.5 to 2.0 mg/ml) of GtnSH and GtnMI media using DMEM-HG. After 24 h of cell seeding, we treated polymer media to HDFs and cultured for 24 h. Next, we treated 10% WST-1 solution to HDFs and incubated for 20 min. The absorbance of the suspension was measured at 450 nm using a microplate reader. The cell viability of the polymers was calculated as a percentage of the control cells (untreated cells, TCPS). To determine the cytotoxicity of the biosealant, we performed a WST-1 assay as described above. In brief, HDFs (1.0 × 10^4^ cells/well) were seeded in a 96-well plate (SPL Life Sciences, Korea) with 100 μl of DMEM-HG supplemented with 10% (v/v) NBCS and 1% (v/v) P/S at 37 °C in 5% CO_2_. Next, we prepared the biosealant eluates by incubating 40 μl of biosealant droplets with 193 μl of media for 24 h. Next, we treated 100 μl of serially diluted eluates to HDFs and incubated them for 24 h. Then, HDFs were incubated with 10% WST-1 solution for 20 min, and the absorbance was measured at 450 nm. The cell viability of the biosealant was calculated as a percentage of the control cells (TCPS). The quantitative analysis of cell viability was analyzed using a Live/Dead assay kit (Invitrogen). After 24 h of biosealant eluate treatment, we treated 193 μl of 2 μM calcein-AM (the acetomethoxy derivative of calcein) and 4 μM ethidium homodimer-1 (EthD-1) mixtures for 15 min. The cellular morphology was qualitatively confirmed using a Nikon Eclipse Ti-2 fluorescence microscope (Nikon, Japan) at ×40 (low magnification) and ×100 (high magnification).

### In vivo biodegradability and tissue compatibility of biosealant

To evaluate the biodegradability of the biosealant, we subcutaneously injected the biosealant solution into mice (6-week-old female C57BL/6N mice) using a dual syringe kit in vivo. All polymers, reagents, and solutions were sterilized by UV irradiation for 15 min or filtered through a syringe filter (pore size: 0.2 μm). After a week of stabilization, the mice were anesthetized with isoflurane in balanced oxygen. Next, we shaved dorsal hair and cleaned their skin with 70% ethanol and povidone. We prepared 200 μl of biosealant solution and subcutaneously injected it into mice (6-week-old female C57BL/6N mice) using a dual syringe kit in vivo. For 8 weeks, we monitored the change in biosealant volume and harvested the biosealant with the surrounding tissues from the mice. The tissue compatibility of the biosealant was evaluated using subcutaneous implantation and H&E staining. After 8 weeks of subcutaneous injection, we harvested various organs and fixed them using 10% formalin. The fixed organs were dehydrated in graded ethanol (80% to 100%), embedded in paraffin, and serially sectioned using a microtome (4 μm). The tissue slides were stained with H&E and observed using a light microscope (Leica DM1000, Leica, Germany) at ×200 magnification. The animal study was performed according to a protocol approved by the IACUC of the Incheon National University (INU-ANIM-2022-02).

### Acute MI model of rats and transplantation of S_3DP in MI model in vivo

All animal experiments and surgical procedures were approved by the IACUC of the Samsung Medical Center (SMCIACUC2021-03-23-001). Male Sprague–Dawley rats (8 to 10 weeks old, 200 to 250 g) were anesthetized with isoflurane gas, endotracheally intubated, and ventilated with air using a small-animal ventilator (Harvard Apparatus, Hopkinton, MA, USA). The S_3DP cultured in a 96-well plate was prepared in a 5% CO_2_ incubator at 37 °C during rat MI modeling. After opening the chest, MI was induced by permanent ligation of the left anterior descending coronary artery (LAD) using 6-0 polypropylene. Ischemia of the anterior wall of the left ventricle (LV) was confirmed by the myocardium turning red to pale pink, and the animals were randomly divided into 5 groups (*n* = 5 per group). The sample codes and compositions are described in Table 2. Before transplanting S_3DP into the heart of the rat, pericardial fluid was removed using sterilized gauze, and the medium in the well containing S_3DP was carefully removed by pipetting to prevent the loss of spheroids from the S_3DP. Then, S_3DP was located on the ischemic heart surface and immobilized using the biosealant. The biosealant was applied along the outside of the 3D patch and allowed to stabilize for 3 min. After confirming that the biosealant and the 3D patch were fixed to the surface of the heart, the chest cavity was closed using 4-0 surgifit sutures. The chest muscles were then closed using 6-0 polypropylene, and the skin was sutured using 6-0 black silk sutures.

### Echocardiography

The rats were anesthetized after 28 days of MI and S_3DP transplantation, and echocardiography was performed for cardiac function evaluation using a VisualSonics Vevo 2100 (VisualSonics Inc., Toronto, ON, Canada). All echocardiography measurements were performed by a single-blind investigator. The left ventricular internal dimension at end-diastole (LVIDd) and left ventricular internal dimension at end-systole (LVIDs) were measured using the 2-dimensional (2D) targeted M-mode to calculate the left ventricular ejection fraction (LVEF) and left ventricular fractional shortening (LVFS). LVEF and LVFS were calculated using the following equations [4,11,12]:

$$\begin{array}{c} \text{LVEF}=\left[\left({\text{LVIDd}}^{3}-{\text{LVIDs}}^{3}\right)\;/{\text{LVIDd}}^{3}\;\times \;100\%\right] \end{array}$$ (1)

$$\begin{array}{c} \text{LVFS}=\left[\left(\text{LVIDd}\;-\;\text{LVIDs}\right)/\text{LVIDd}\;\times \;100\%\right] \end{array}$$ (2)

### Histological analysis

After echocardiography, whole hearts were harvested from the sacrificed rats and fixed with 10% formalin. The hearts were sliced into 3 sections along the transverse axis below the ligature to the apex at 2-mm intervals. The samples were dehydrated in a series of ethanol, embedded in paraffin, and cut into 4-μm-thick sections. The sections were deparaffinized in xylene, ethanol, and distilled water and stained with H&E and Masson’s trichrome to visualize cardiac fibrosis. Stained slides were scanned using the Aperio ScanScope AT Slide Scanner (Leica Biosystems Inc., Buffalo Grove, IL, USA) at ×200 magnification, and all images were captured using ImageScope software (Leica Biosystems Inc.). The infarction size and fibrosis area of the LV were determined using the following formula [13]:

$$\begin{array}{c} \text{Infarction size}=\\ \left[\left(\text{epicardial circumference of the total LV area}\;\right.\right.\\ +\;\left.\text{endocardial circumference of the total LV area}\right)/\\ \left(\text{epicardial circumference of the total LV area}\right.\\ \left.\left.+\;\text{endocardial circumference of the total LV area}\right)\;\times \;100\%\right] \end{array}$$ (3)

$$\begin{array}{c} \text{Fibrosis area}\;=\;\left[\left(\text{fibrosis area of LV}/\text{total area of LV}\right)\;\times \;100\%\right] \end{array}$$ (4)

The LV wall thickness was measured in the infarcted and border zones. Each zone was divided into 6 equal segments, and LV wall thickness was calculated by averaging the thicknesses of the 6 segments [6]. All histological analyses were quantified using ImageJ software.

### Statistical analysis

The GraphPad Prism 5 and 7 software (GraphPad Software, San Diego, CA, USA) was used for all statistical analyses. Statistical analysis was performed using a *t* test and one-way analysis of variance (ANOVA) with the Bonferroni test, and all quantitative data were presented as mean ± standard deviation (SD). Statistical significance was set at *P* < 0.05.

## Results and Discussion

### Fabrication of open and open/closed pocket patches

**Fig. 2.**
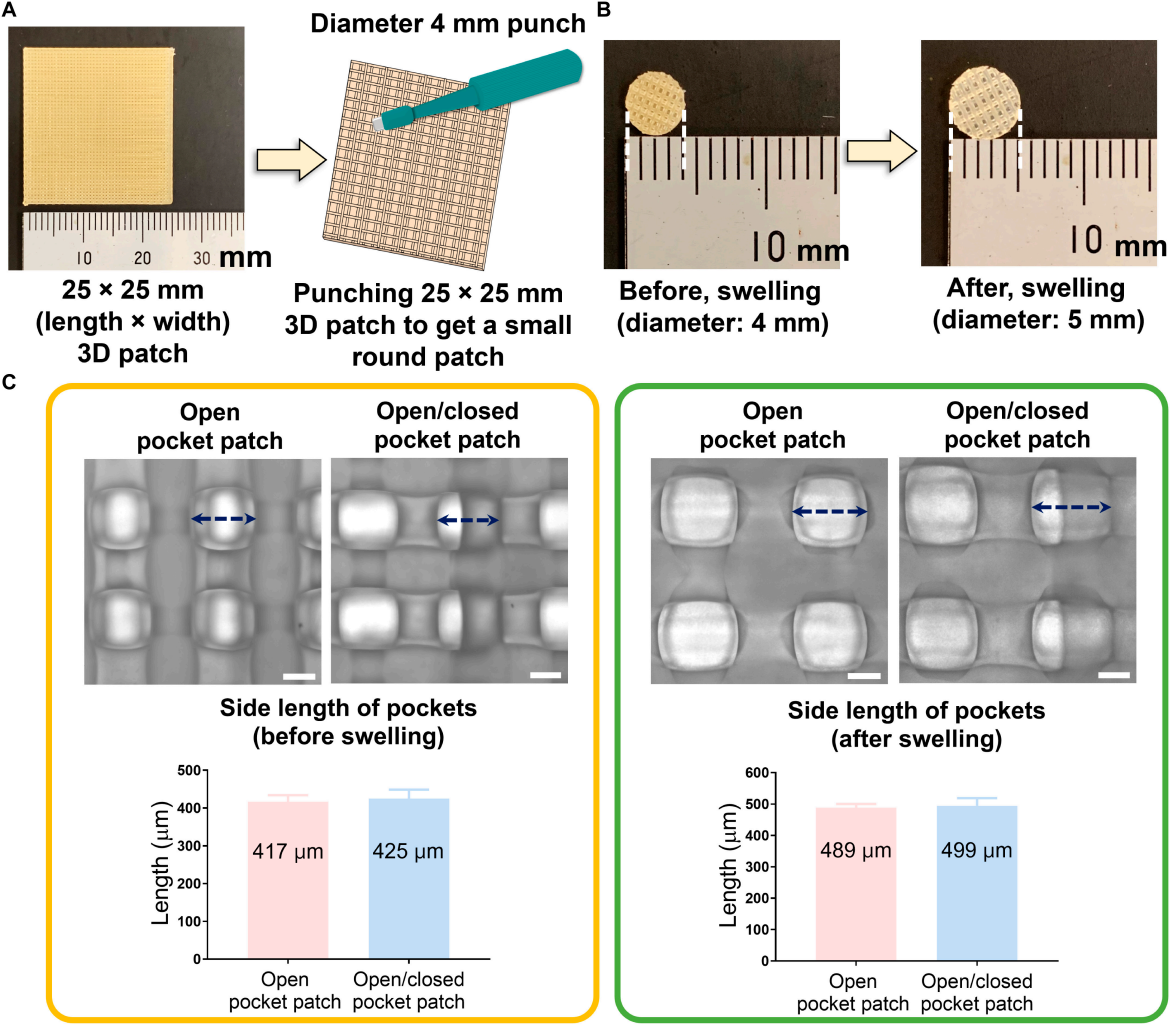
Characteristics and preparation of the 3D patches for hADSC seeding. (A) The photograph of the 25 × 25 mm 3D patch and an illustration of 3D patch preparation for the experiments. (B and C) Swelling characteristic of the 3D-printed patch. The photographs, micrographs, and graphs of the open pocket patch and open/closed pocket patch before and after swelling. The result in (C) is shown as the average values ± SD (*n* = 24). Scale bar: 200 μm.

In our previous study, we synthesized TPU-CEC363 using PEG, ε-caprolactone, and iron (III) acetylacetonate to develop a 3D-printable patch with dual pockets [28]. TPU-CEC363 has elasticity, flexibility, thermal property, water uptake, and biodegradability, enabling the patch to withstand repeated contractions and relaxations of the heart. We punched a patch with a diameter of 4 mm to obtain a uniform shape (Fig. 2A). In the swelling test, the 3D patches swelled owing to the hydrophilic properties of the TPU-CEC363. We confirmed that the diameter of the patches increased slightly from 4 to 5 mm after swelling (Fig. 2B). We randomly measured the side lengths of the open and closed pockets to confirm differences in pocket size after swelling. As a result, the side length of the pockets was similarly increased by approximately 70 μm after swelling, and there was no significant difference between open and closed designs (Fig. 2C). These results indicate that we successfully fabricated the 3D-printed patch and constructed uniform dual pocket structures under moist conditions. Furthermore, the 3D-printed patches provided a uniform and sufficient cavity after swelling for spheroid formation.

### Fabrication and characterization of hADSC spheroids in open and open/closed pocket patches

**Fig. 3.**
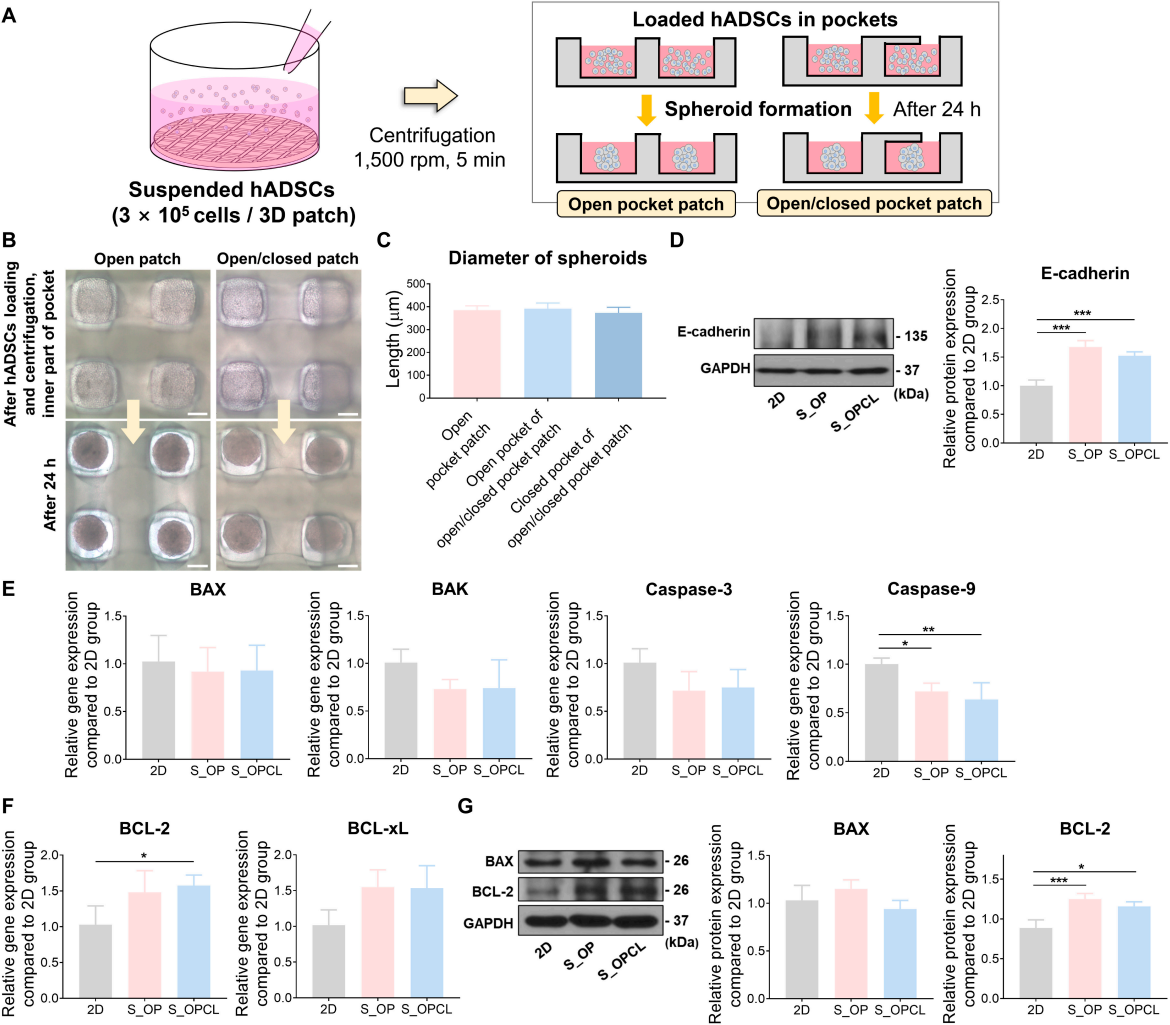
Morphology and viability of hADSC spheroids in the open pocket and open/closed pocket patches. (A) Schematic illustration of the hADSC spheroids fabrication. (B) Representative optical images of hADSCs within the 3D patches and spheroids after 24 h. Scale bar: 200 μm. (C) The average diameter of spheroids formed in the 3D patches. The result in (C) is shown as the average values ± SD (*n* = 16). (D) Western blot analysis and quantification data comparing E-cadherin between 2D cells and spheroid within 3D patches. The gene expression of (E) apoptotic factors (BAX, BAK, Caspase-3, and Caspase-9) and (F) anti-apoptotic factors (BCL-2 and BCL-xL). (G) Western blot analysis of the representative apoptotic factor (BAX) and anti-apoptotic factor (BCL-2). The results in (D) and (G) are shown as the average values ± SD (*n* = 3). * indicates a statistical significance compared to the 2D group (*P < 0.05 and ***P < 0.005). The results in (E) and (F) are shown as the average values ± SD (*n* = 4). * indicates a statistical significance compared to the 2D group (*P < 0.05 and **P < 0.01).

To fabricate the stem cell spheroid within the 3D-printed patches, we optimized the number of hADSCs with the open/closed pocket patch. We loaded suspensions from 2 × 10^5^ to 5 × 10^5^ cells onto the 3D-printed patches and centrifuged them to enter the cells within pockets (Fig. 3A and Fig. S1A). After 24 h of cell seeding, we confirmed the morphology of the formed spheroids according to the cell number (Fig. S1A).

When 2 × 10^5^ cells were loaded, insufficient cells were loaded in the closed pockets, forming small-sized spheroids (Fig. S1A and B). At 3 × 10^5^ cells, we observed that spheroids with uniform morphology were formed in the pockets (Fig. S1A and Fig. 3B). The size of the spheroids did not significantly differ depending on the pocket type (Fig. S1B and Fig. 3C). However, above 3 × 10^5^ cells, the restricted entrance size of the closed pockets inhibited the homogeneous distribution of cells in both the open and closed pockets. As a result, the size of the spheroids in the open pockets gradually increased as the number of cells increased (Fig. S1A and B). The focus is on uniformly loading the cells into the fabricated 3D patches regardless of pocket type.

Next, we evaluated the viability of spheroids and their expression of angiogenic growth factors depending on cell numbers. The gene expression of BCL-2, an anti-apoptotic factor, was highest in spheroids formed with 3 × 10^5^ cells (Fig. S1C), and the gene levels of apoptotic factors (BAX and Caspase-3) showed relatively high expression in spheroids formed with 2 × 10^5^ cells (Fig. S1D). Furthermore, the gene expression of VEGF was not significantly different depending on the cell number, but the gene expression patterns of HGF and fibroblast growth factor (FGF)-2 were different (Fig. S1E). Nevertheless, the gene expressions of spheroids formed by 3 × 10^5^ cells were relatively higher than those of other groups for both factors. Therefore, we chose the 3 × 10^5^ cells/patch as the appropriate cell number for the open/closed pocket patch and evaluated the treatment efficacy through comparison with 2D cells.

During spheroid formation, it is well known that cell-to-cell interactions increase with the accumulation of cadherin protein on the cell membrane. In particular, E-cadherin plays a key role in spheroid formation, and cadherin-to-cadherin binding compacts cell aggregation to form spheroids [31–33]. Therefore, we evaluated the effect of the pocket system on spheroid formation by analyzing E-cadherin expression. The protein expression of E-cadherin was increased in the spheroid-laden open pocket patch (S_OP) and spheroid-laden open/closed pocket patch (S_OPCL) groups compared to that in the 2D group because the pocket system structurally induced the aggregation of hADSCs, increasing cell-to-cell interactions (Fig. 3D).

In fabricating a spheroid, the size of a spheroid is crucial in determining its viability because it impacts oxygen and nutrient supply within the spheroid. If the spheroid size is >500 μm, the inner core accumulates the toxic metabolic waste and forms a necrotic core [31,33]. Therefore, we examined spheroid viability using qRT-PCR and Western blotting. Compared to the 2D group, the gene expression of apoptotic factors, such as BAX, Bcl-2 homologous antagonist killer (BAK), and Caspase-3, tended to decrease in the S_OP and S_OPCL groups. Moreover, the expression of Caspase-9 was significantly lower in these groups (Fig. 3E). Cell apoptosis is initiated by sequential activation of several caspases. Among various caspases, Caspase-9, an initiator caspase, is activated by forming an apoptosome with cytochrome c and apoptotic protease activating factor-1 (Apaf-1). The activated Caspase-9 stimulates Caspase-3, an effector caspase, to initiate cell apoptosis [34,35]. In contrast, the gene expression of anti-apoptotic markers, such as BCL-2 and B-cell lymphoma extra-large (BCL-xL), in the S_OP and S_OPCL groups tended to be higher than those in 2D cells (Fig. 3F). Furthermore, regarding protein expression, we confirmed that BCL-2 expression was significantly increased in the S_OP and S_OPCL groups, whereas BAX expression was not significantly different from that in 2D cells (Fig. 3G). Consequently, our 3D patches with a dual module pocket demonstrated their potential as a culture system for fabricating stem cell spheroids without cytotoxic issues.

### Paracrine angiogenic effect of S_3DP

Stem cell spheroids have been reported to exhibit superior therapeutic effects compared with conventional monolayer stem cells because of their upregulated paracrine secretion [11,32]. Previous studies reported that the expression of hypoxia-inducible factor 1-alpha (HIF-1α) increases in the mildly hypoxic inner zone of spheroids [31,32]. HIF-1α acts as a transcription factor and regulates various target genes related to angiogenesis [32]. With this rationale in mind, we compared the angiogenesis-related factor expression and paracrine effects between our system and 2D cultured stem cells in vitro.

**Fig. 4.**
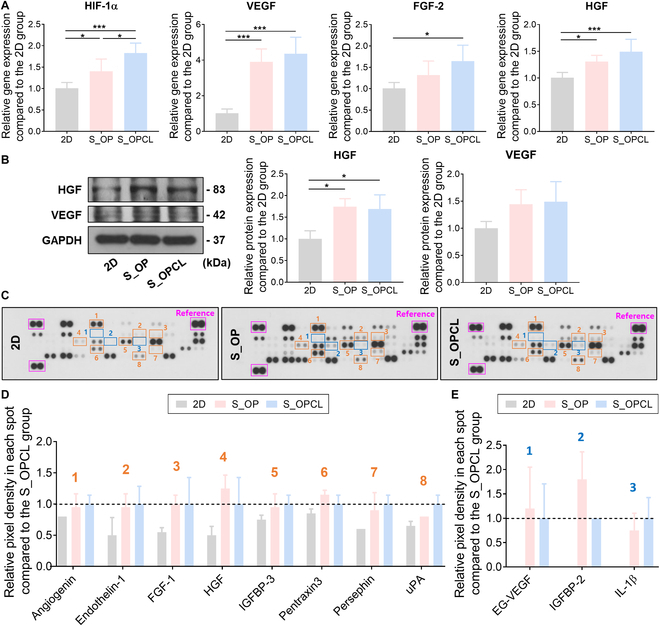
Paracrine effect of angiogenic factors of hADSC spheroids in the open pocket and open/closed pocket patches. The relative expression of HIF-1α and angiogenic growth factors (VEGF, FGF-2, and HGF) of the 2D group and experimental groups was evaluated using (A) qRT-PCR and (B) Western blot analysis. (C) Representative images of the human angiogenesis antibody array data of the 2D group and the experimental groups. (D) The relative expression of angiogenesis-related proteins in CM. (E) The relative expression of angiogenic proteins that were only expressed in the experimental groups, not in the 2D group. The results in (A) and (B) are shown as the average values ± SD (*n* = 5 and 3). * indicates a statistical significance compared to each group (*P < 0.05 and ***P < 0.005). The results in (D) and (E) are shown as the average values ± SD (*n* = 2).

We first evaluated gene expression of HIF-1α, resulting in the S_OP and S_OPCL groups upregulated gene expression of HIF-1α compared to the 2D group (Fig. 4A). Next, we analyzed the expression of VEGF, FGF-2, and HGF as representative pro-angiogenic markers [9,31]. The S_OP and S_OPCL groups showed increased gene expression of each factor compared to that in the 2D group (Fig. 4A). Notably, the gene expression of VEGF was significantly increased by more than 3 times. Following gene expression analysis, we performed Western blotting to assess the protein expression of angiogenesis-related growth factors, such as HGF and VEGF (Fig. 4B). The levels of both growth factors were higher in the S_OP and S_OPCL groups than those in the 2D group. Based on these results, our spheroid-laden patch system promotes the expression of growth factors related to angiogenesis.

hADSCs secrete a wide range of factors that promote angiogenesis, reduce apoptosis, and regulate inflammatory responses. The paracrine effects of hADSCs have been validated in numerous studies of MI treatment [5,31]. Consequently, the secretome released from hADSCs is considered a promising source for cytokine-based therapy, and its secretion and angiogenic potential have been reported to be promoted under 3D culture conditions [15,31,33]. To investigate whether our 3D culture system could induce a stronger paracrine effect by releasing increased angiogenesis-related factors compared to 2D culture, we analyzed the collected CM using an angiogenesis array kit. The expression of various angiogenic factors was increased in the CM obtained from the S_OP and S_OPCL groups compared to that in the CM from the 2D group (Fig. 4C). Notably, pro-angiogenic factors such as angiogenin, endothelin-1, FGF-1, HGF, insulin-like growth factor-binding protein (IGFBP)-3, pentraxin-3, persephin, and urokinase-type plasminogen activator (uPA) were modestly increased in the S_OP and S_OPCL groups compared to those in the 2D group (Fig. 4D). Interestingly, endocrine gland-derived vascular endothelial growth factor (EG-VEGF), IGFBP-2, and interleukin-1 beta (IL-1β) were detected only in the S_OP and S_OPCL groups, but not expressed in the 2D group (Fig. 4E). These proteins have the potential angiogenic capacity, and their expression is known to be regulated by HIF-1α [36–39]. In particular, EG-VEGF and IL-1β are influenced by the expression of HIF-1α because they have HIF-1α binding sites in their promoter regions [40,41]. Furthermore, IGFBP-2 acts as a transcriptional enhancer that promotes the activity of the VEGF gene promoter through nuclear translocation, leading to enhanced VEGF expression [36,37]. Consequently, we speculate that the increased expression of HIF-1α within the hypoxic core of the spheroids may stimulate the expression of EG-VEGF, IGFBP-2, and IL-1β, resulting in the paracrine angiogenic effect of S_3DP.

Based on these, the cytokines expressed only in the CM of the S_OP and S_OPCL groups showed more marked therapeutic potential than conventional 2D cell transplantation. Consequently, we demonstrated that our system could improve the secretion of various angiogenic factors and their paracrine effects through CM.

### Fabrication and characterization of tissue adhesive biosealant

**Fig. 5.**
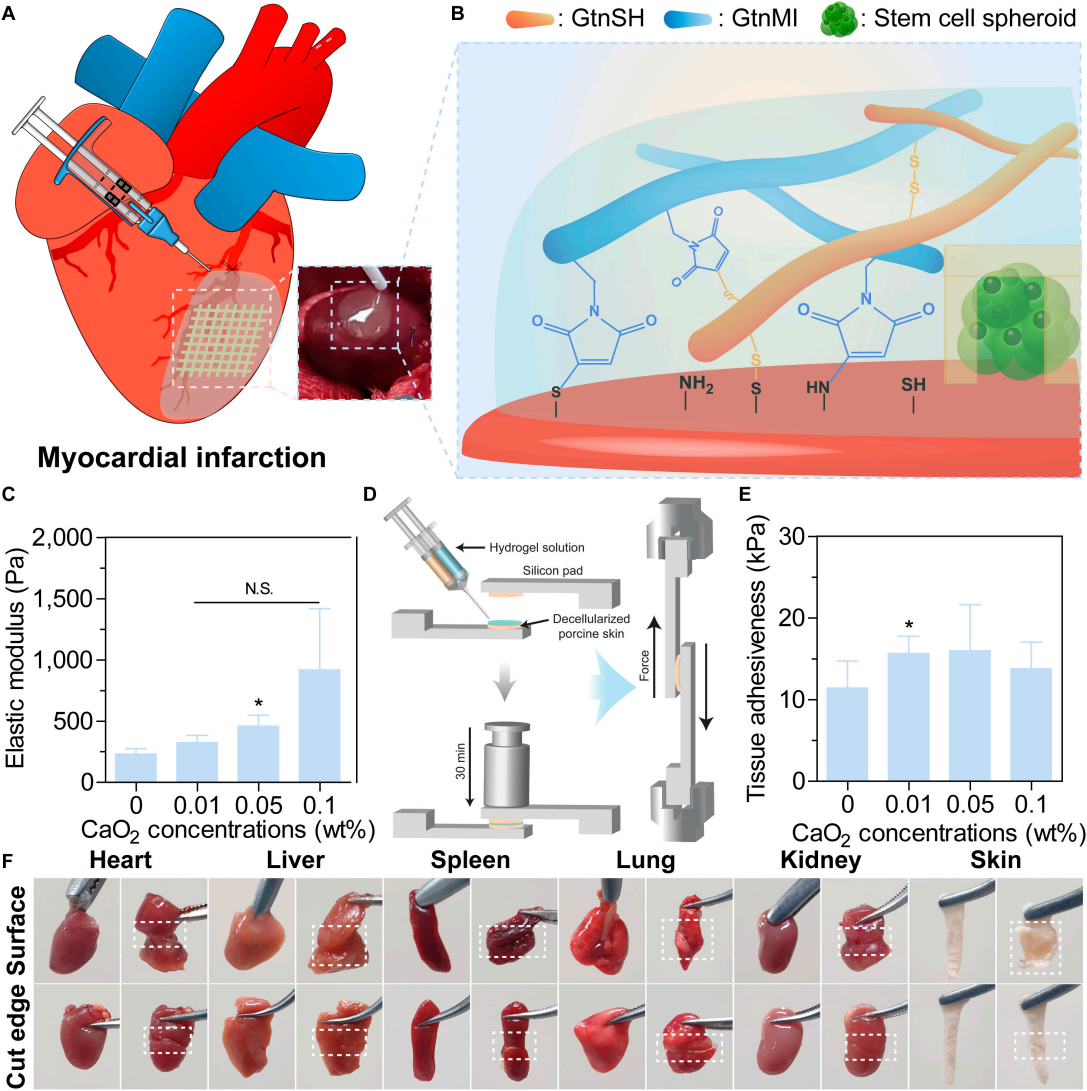
Fabrication and characterization of biosealant. (A) Schematic illustration of biosealant for fixing S_3DP. (B) Mechanism of biosealant formation and tissue adhesion. (C) Mechanical property of biosealant with various CaO_2_ concentrations. The result in (C) is shown as the average values ± SD (*n* = 3). * indicates a statistical significance compared to CaO_2_ 0 wt% (*P < 0.05). (D) Schematic illustration of tissue adhesiveness. Tissue adhesiveness of biosealant on (E) decellularized porcine skin and (F) various organ surfaces. The result in (E) is shown as the average values ± SD (*n* = 6). * indicates a statistical significance compared to CaO_2_ 0 wt% (*P < 0.05).

To immobilize S_3DP on the heart surface, we developed a gelatin-based biosealant via the dual crosslinking reactions involving the thiol-ene reaction and disulfide bond formation (Fig. 5A and B). In this system, the biosealant is first crosslinked via a thiol-ene reaction between the functional groups, thiol and maleimide groups, in the polymers. We used CaO_2_ as an additional crosslinker to reinforce the mechanical stiffness of the biosealant. CaO_2_ and its derivative, calcium hydroxide (Ca(OH)_2_), have been extensively used in food, cosmetic, and biomedical applications [29]. Notably, it is well known that CaO_2_ produced hydrogen peroxide (H_2_O_2_) during its decomposition in aqueous media, inducing disulfide bond formation via H_2_O_2_-mediated thiol oxidation. Furthermore, these chemical reactions of the hydrogel enabled attachment to the heart via the interaction of maleimide and thiol groups with the primary amine and thiol groups on the heart surface. Therefore, GtnSH and GtnMI were synthesized as polymers of biosealant using the EDC/NHS chemistry (Fig. S2A). Gtn was selected as the polymer backbone owing to its biocompatibility, biodegradability, bioactivity, and easy modification [29]. Next, we characterized the chemical structures of GtnSH and GtnMI by ^1^H-NMR spectroscopy. As a result, GtnSH exhibited new peaks at 2.8 and 3.5 ppm, indicating the conjugation of cystamine to the carboxyl group of Gtn [42] (Fig. S2B). For GtnMI, we confirmed a sharp peak appearance at 6.8 ppm and a decreased peak area at 3.0 ppm, which indicates the introduction of the maleimide group to a primary amine group of Gtn [30,43] (Fig. S2C). Next, we determined the functional groups of GtnSH and GtnMI using Ellman’s assay. GtnSH and GtnMI revealed 158.3 ± 42.7 and 87.8 ± 13.9 μmol/g of polymers, respectively. Based on these results, we successfully synthesized polymers for fabricating tissue adhesive biosealant.

The viscoelasticity of the biosealant is crucial for patch fixation and sealing, particularly in resisting external forces, such as the heartbeat. Accordingly, we fabricated tissue adhesive biosealants with various CaO_2_ concentrations and measured their *G*′ using a rheometer. As increasing CaO_2_ concentration, the *G*′ of hydrogels was reinforced (0 wt%, 230 Pa; 0.01 wt%, 360 Pa; 0.05 wt%, 420 Pa; 0.1 wt%, 920 Pa) (Fig. 5C). Typically, the stiffness of the rat myocardium is known to range from 0.1 to 140 kPa [44]. Additionally, it has been reported that the optimal *G*′ value of the hydrogel matrix for cardiac repair ranges from 380 to 600 Pa, representing a relatively soft matrix. This soft matrix facilitates the transmission of mechanical signals and the coordination of myocardial tissue, improving the reconstruction of cardiac function [45]. Consequently, we controlled the stiffness of our biosealant using CaO_2_ concentrations, providing a suitable matrix in the dynamic cardiac environment for effective cardiac repair.

The tissue adhesive force of the biosealant should withstand external forces such as heartbeat and blood pressure and stably immobilize the patch in place. Therefore, we measured the tissue adhesive force of our biosealant at various CaO_2_ concentrations using decellularized porcine skin and a UTM (Fig. 5D). We observed that CaO_2_ addition reinforced the tissue adhesive strength of the biosealant compared to that of the control (CaO_2_ 0 wt%, 11.3 kPa; 0.01 wt%, 15.7 kPa; 0.05 wt%, 15.4 kPa; 0.1 wt%, 14.0 kPa) (Fig. 5E). These tissue adhesive strengths were higher than commercially available fibrin glue [46,47] (5 to 10 kPa). Our biosealant could strongly attach to tissue surfaces through a thiol-ene reaction and disulfide bond formation, in contrast to fibrin glue, which relies on hydrogen bond formation during coagulation [48] (Fig. 5B). Interestingly, we confirmed that the increasing tissue adhesive strength was saturated between CaO_2_ 0.01 and 0.05 wt% and decreased at CaO_2_ 0.1 wt%. The number of tissue adhesive functional groups, such as thiol groups, and gelation time were rapidly decreased when the CaO_2_ concentration was increased, resulting in insufficient tissue adhesive groups and time to interact with the tissue surface. Accordingly, we selected CaO_2_ 0.1 wt% as the optimal composition of biosealant for immobilizing a patch in vivo, homogeneously.

Next, we determined the feasibility of biosealant on various organ surfaces (Fig. 5F). Interestingly, our sealant bonded between tissue surfaces and cut areas of various organs. The tissue adhesion of our sealant targets the thiol and primary amine groups present on most tissue surfaces. This result suggests that our biosealant can be used as an adhesive to fix patches on various tissue surfaces.

Consequently, we developed gelatin-based tissue adhesive biosealants with tunable elasticity and stronger tissue adhesiveness than commercial fibrin glue. This biosealant has great potential as an advanced sutureless technique to immobilize implantable devices in vivo.

### Biocompatibility and biodegradability of biosealants

**Fig. 6.**
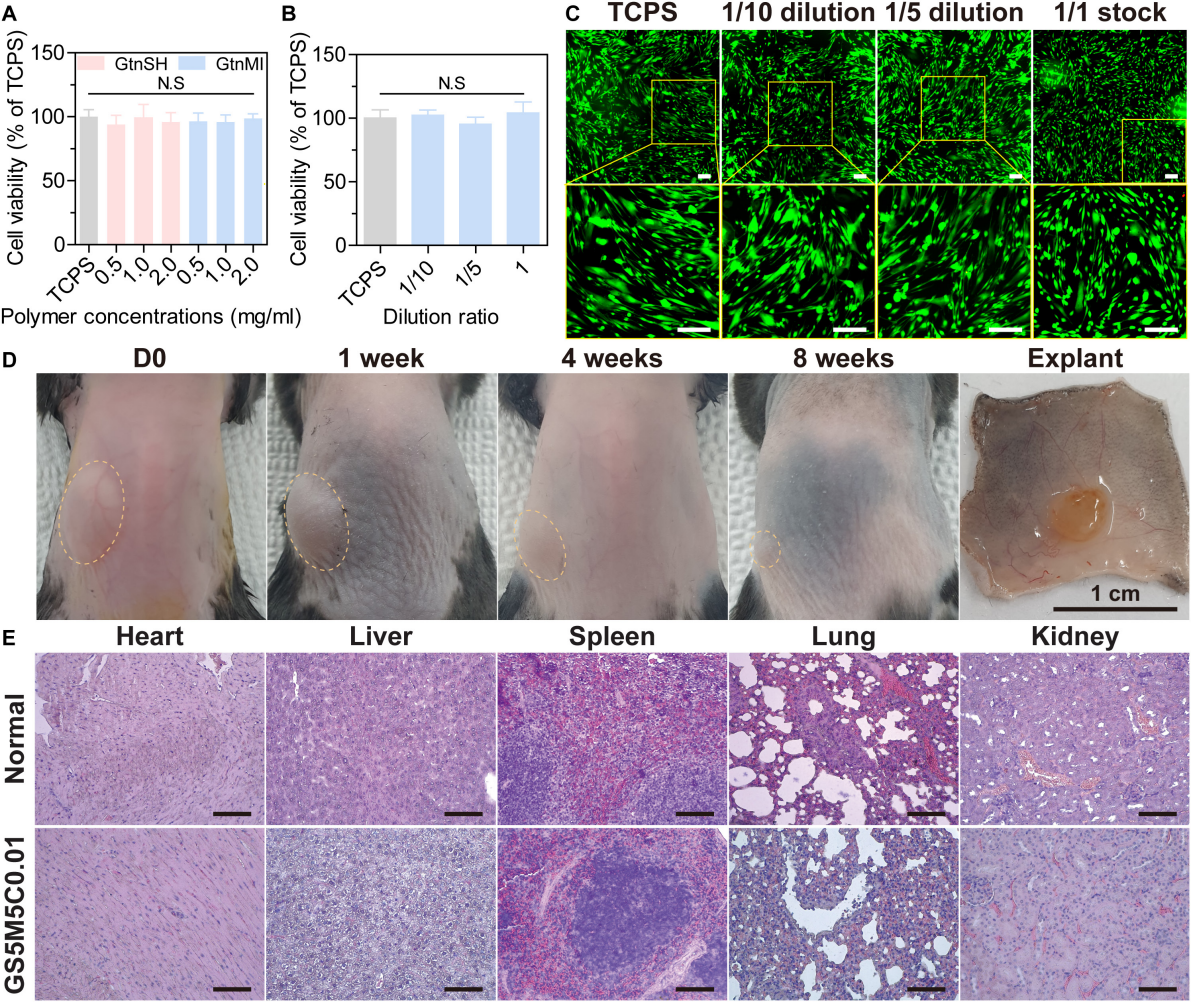
Biocompatible and biodegradable biosealant. Cytocompatibility of (A) polymer and (B) biosealant on HDFs. (C) Fluorescence images of the HDFs after a day of hydrogel eluate treatment. (D) Biodegradability and (E) tissue compatibility of biosealant in vivo. The results in (A) and (B) are shown as the average values ± SD (*n* = 6). Scale bar: 100 μm.

The biocompatibility and biodegradability of biosealant are essential properties for successful clinical applications because they ensure the safety of materials and their by-products and suitable maintenance of the sealant at a rate compatible with tissue healing [22]. To utilize our sealant for immobilizing the S_3DP in vivo, we first evaluated the cytocompatibility of the polymers using HDFs and the WST-1 assay in vitro. GtnSH and GtnMI showed high cell viability above 94% in TCPS (Fig. 6A). This result indicates that our biosealant may be cytocompatible with host cells. Additionally, the biosealant exhibited excellent cell viability above 95.2% of TCPS and the predominant viable cell population (Fig. 6B and C).

We next subcutaneously injected the biosealant in mice to evaluate its biodegradability and tissue compatibility in vivo. Figure 6D shows that the sealant was maintained for 8 weeks and was sustainably degraded in vivo. This suggests that our sealant is biodegradable and can be maintained at a specific volume within the body for 8 weeks in vivo. Furthermore, we evaluated the tissue compatibility of the biosealant using various major organs harvested 8 weeks after subcutaneous injection. Compared with normal organs, our biosealant and their by-products revealed no pathological changes in the major organs in vivo (Fig. 6E).

Consequently, our biosealant proved to be biocompatible and biodegradable with the potential to fix patches in vivo. Based on these results, we investigated the therapeutic effects of the S_3DP on cardiac infarction.

### The therapeutic effect of S_3DP on cardiac infarction

**Fig. 7.**
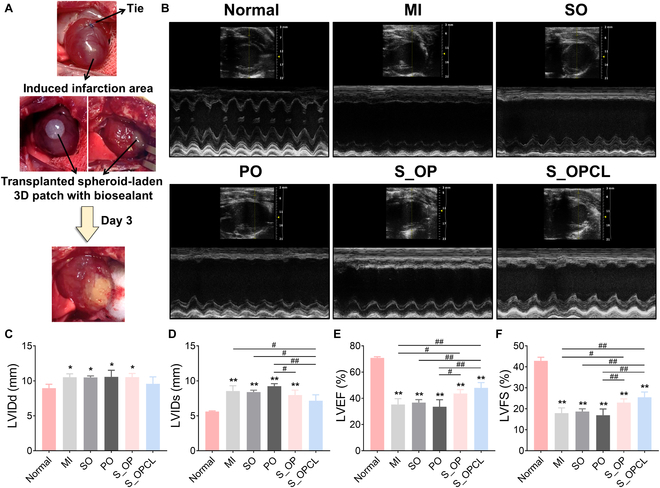
Transplantation of S_3DP in the rat MI model and improved cardiac function. (A) Representative photographs of the heart after acute MI modeling and transplantation of S_3DP with biosealants. (B) Representative echocardiographic images of each group 4 weeks after S_3DP transplantation. The (C) LVIDd, (D) LVIDs, (E) LVEF, and (F) LVFS were assessed using 2D echocardiography. The results in (C) to (F) are shown as the average values ± SD (*n* = 5). * and # indicate a significant difference compared to the normal group and each group (*P and ^#^*P* < 0.05, **P and ^##^*P* < 0.005).

We investigated the therapeutic efficacy of S_3DP using a rat MI model. To fabricate the MI model, we tied the LAD to induce permanent vascular occlusion and observed changes in myocardial color [7]. After fabricating the MI model, the S_3DP was immediately immobilized on the heart surface using our biosealant and stabilized for 3 min. As a result, we successfully attached the patch to the heart surface without slippage under a dynamic heartbeat, and it was stably maintained for 3 days (Fig. 7A). An additional movie file shows this in more detail (Movie S1). To compare the effect of patches or spheroids on MI treatment, we selected the sealant-only (SO) and open/closed pocket patch-only (PO) groups for comparison. After 4 weeks post-transplantation, we examined LVEF and LVFS, which are the most commonly used parameters to quantify heart function using echocardiography [3,11,49] (Fig. 7B). We first measured LVIDd and LVIDs to calculate the LVEF and LVFS using the photographed ultrasound results [49]. LVIDd and LVIDs in the controls (MI, SO, and PO) and S_OP group were notably higher than those in the normal group. However, there was no significant difference in the results of LVIDd and LVIDs between the S_OPCL and normal group. Moreover, the S_OPCL group showed significantly decreased LVIDs compared to the control groups (Fig. 7C and D). LVIDs is the result of myocardial contractility, meaning that the narrower the LVIDs, the better the heart function. This result demonstrated that the delivery of hADSC spheroids effectively improved myocardial function through their paracrine effect, and open and closed pockets were more suitable than open pockets.

LVEF and LVFS were calculated using established formulas, with the internal diameter values measured during systole and diastole. LVEF is the fraction of blood ejected by the LV during the cardiac cycle, and LVFS indicates the percentage change in the LV internal dimensions between systole and diastole [3]. All groups showed lower heart function than the normal group in terms of both LVEF and LVFS. However, we confirmed that the S_3DP groups (S_OP and S_OPCL) had significantly improved cardiac functions with increased LVEF and LVFS compared to the control groups (Fig. 7E and F). Consequently, we successfully immobilized our S_3DP system using a biosealant without physical damage, which occurs by suturing or injection. Our system was stably maintained for 4 weeks even in the presence of pericardial fluid. Moreover, we demonstrated its therapeutic effects in the restoration of cardiac function. Notably, the combination of open and closed pockets was the most effective through the paracrine effect and protection of the spheroids against harsh infarcted conditions.

### Reduced cardiac fibrosis of S_3DP on cardiac infarction

**Fig. 8.**
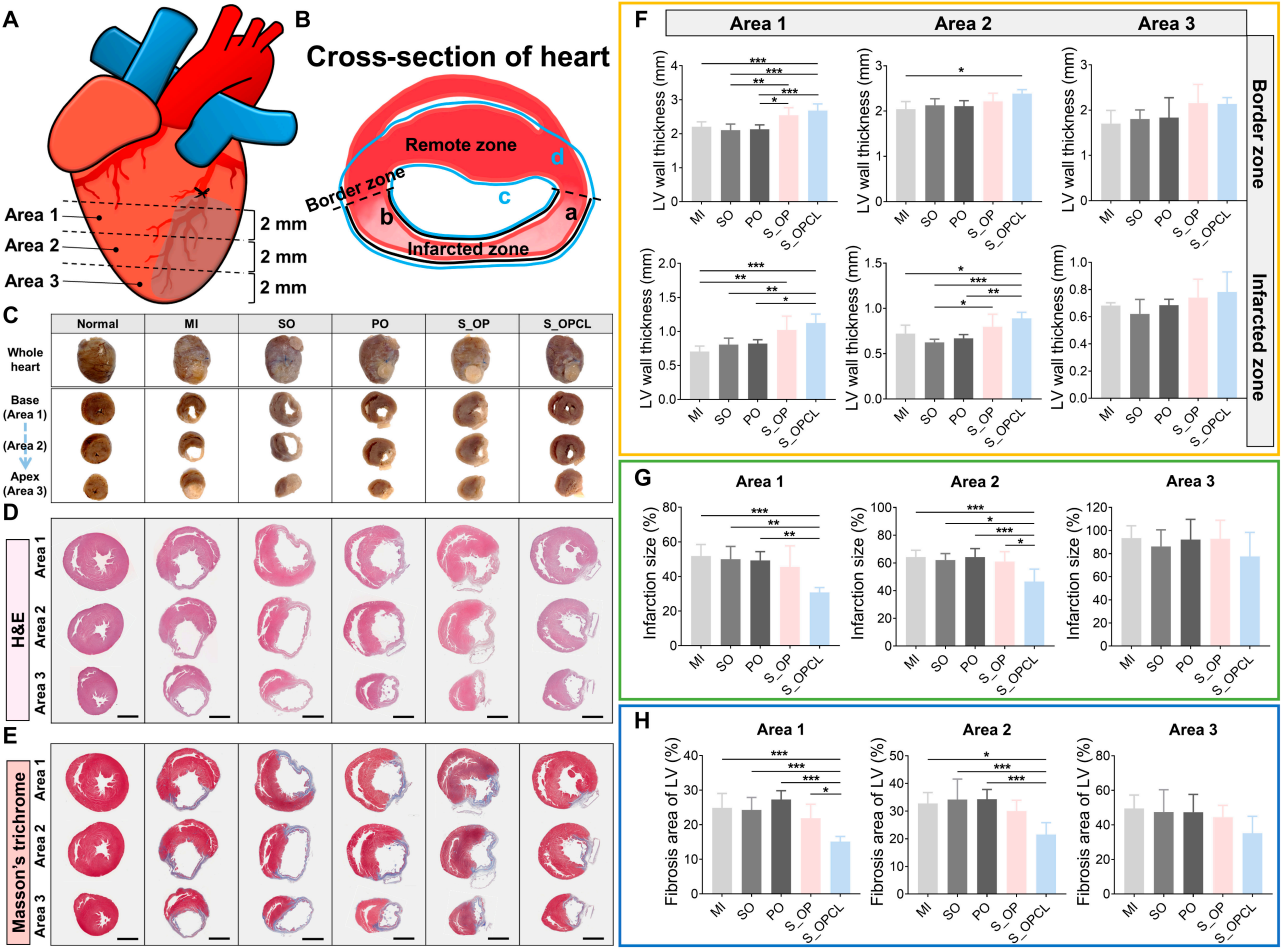
Therapeutic effect of the S_3DP on reducing cardiac fibrosis. Schematic illustration showing (A) the location of areas 1 to 3 in the heart and (B) the cross-section of the heart. (C) Representative images of the whole heart and each area. Representative (D) H&E and (E) Masson’s trichrome stained images of areas 1 to 3 after 4 weeks of surgery and treatment. Scale bar: 4 mm. Quantification of (F) the wall thickness, (G) infarction size, and (H) fibrosis area of the LV in each area based on Masson’s trichrome staining. The results in (F) to (H) are shown as the average values ± SD (*n* = 5). * indicates statistical significance compared to each group (*P < 0.05, **P < 0.01, ***P < 0.005).

Following MI, cardiomyocytes undergo cell death while cardiac fibroblasts are activated, replacing the damaged myocardium with collagen-based scar tissue [11,50]. However, this fibrotic tissue cannot perform normal functions, such as contraction and relaxation. Thus, reducing the fibrotic area in the LV is important for maintaining or restoring cardiac function. MI is accompanied by ECM degradation, LV wall thinning, and ventricular enlargement [10,50]. Based on these, we histologically analyzed LV wall thickness, infarction size, and fibrosis area. We sectioned the whole heart into 3 areas at 2-mm intervals from the LAD-tied part, which are indicated in areas 1 to 3 (Fig. 8A and C). We then divided the cross-sectional tissue of the heart into 3 zones: an infarcted zone (IZ), a border zone (BZ), and a remote zone (RZ). We focused our analysis on the IZ and BZ [7] (Fig. 8B). To determine the fibrotic area of the heart, we performed H&E and Masson’s trichrome staining on sectioned tissues (Fig. 8D and E). The blue area indicates fibrotic tissue (collagen), and the red area indicates the myocardium (Fig. 8E).

During post-infarction repair, fibrotic tissue formation causes progressive thinning of LV wall thickness [50]. Therefore, we first measured the LV wall thickness according to the zone. In area 1, the LV wall thicknesses in the S_3DP groups (S_OP and S_OPCL) were significantly greater than those in the control groups (MI, SO, and PO) in both the BZ and IZ. In area 2, only the MI and S_OPCL groups showed a significant difference in the BZ. Interestingly, in the IZ of area 2, the LV wall thickness in the S_OPCL group was considerably greater than those in the control groups. However, in area 3, no significant differences were observed between all groups (Fig. 8F). Based on these results, we confirmed that the LV wall thickness was increased by the spheroid treatment through S_3DP, except in area 3, and the S_OPCL group was more effective than in the S_OP group.

Furthermore, we analyzed the infarct size and fibrotic area of LV. The following formula: [(*a* + *b*)/(*c* + *d*) × 100%] was used to calculate the infarction size by measuring the length of the letter, as shown in Fig. 8B. There were no differences among the control groups in any area. The infarction size in the S_OPCL group was significantly reduced in areas 1 and 2 compared with those in the control groups, but the infarction size in the S_OP group did not decrease in any area (Fig. 8G). To determine the percentage of fibrosis, we measured the total LV and fibrotic areas in Fig. 8E. There were no significant differences between the control groups in any area. In all areas, the fibrosis area of the S_OP group was decreased compared to those of the control groups, although the differences were not statistically significant. In contrast, the fibrosis area of the S_OPCL group was notably reduced compared to those of all groups in all areas and revealed only significant differences in areas 1 and 2 (Fig. 8H).

In conclusion, we confirmed that S_3DP transplantation effectively reduces fibrotic scar tissue and increases LV wall thickness. Notably, the S_OPCL group showed greater potential therapeutic effects than the S_OP group, which is consistent with the cardiac function results.

## Conclusion

In this study, we developed S_3DP and biosealant as a new type of stem cell spheroid therapy to treat MI. We designed S_3DP with dual pore modules (open and closed pockets) to improve the engraftment rate and paracrine effect of the spheroids. The 3D patches provided uniform conditions for stem cell culture and spheroid fabrication with high cell viability. Moreover, these spheroids produced secretomes related to angiogenesis and exhibited improved paracrine effects in vitro. Next, we fabricated a tissue adhesive biosealant as a sutureless method for immobilizing S_3DP to the heart tissue, maintaining its position for 4 weeks in vivo. When we transplanted our system into a rat MI model, S_3DP facilitated the restoration of cardiac function and decreased the infarcted area. Notably, the combination of open and closed pocket, S_OPCL, was more effective than the open pocket, S_OP, demonstrating that the protective effect of the closed pocket acts synergistically with direct spheroid delivery and paracrine effects. Collectively, our system has great potential as a new type of spheroid delivery system with a sutureless approach for MI treatment and can overcome the challenges of heart disease therapy.

## Data Availability

The data are available from the authors upon a reasonable request.
