# Supplementary material for: Transplantation of Stem Cell Spheroid-Laden 3-Dimensional Patches with Bioadhesives for the Treatment of Myocardial Infarction
Source: Biomater Res. 2024 Mar 4;28:0007. doi: 10.34133/bmr.0007 (PMC10911933; doi:10.34133/bmr.0007)
Supplement: Supplementary 1 — Tables S1 and S2 Figs. S1 and S2 Movie S1 [file bmr.0007.f1.docx]

Supporting materials

**Transplantation of stem cell spheroid-laden three-dimensional patches with bioadhesives for the treatment of myocardial infarction**

Hye Ran Jeon^1,†^, Jeon Il Kang^2,†^, Suk Ho Bhang^3^, Kyung Min Park^2,4*^, and Dong-Ik Kim^1,5*^

^1^Department of Health Sciences and Technology, Samsung Advanced Institute for Health Sciences and Technology (SAIHST), Sungkyunkwan University, Seoul 06355, Republic of Korea. ^2^Department of Bioengineering and Nano-Bioengineering, College of Life Sciences and Bioengineering, Incheon National University, 119 Academy-ro, Yeonsu-gu, Incheon 22012, Republic of Korea. ^3^School of Chemical Engineering, Sungkyunkwan University, Suwon 16419, Republic of Korea. ^4^Research Center for Bio Materials & Process Development, Incheon National University, 119 Academy-ro, Yeonsu-gu, Incheon 22012, Republic of Korea. ^5^Division of Vascular Surgery, Sungkyunkwan University School of Medicine, Samsung Medical Center, Seoul 06351, Republic of Korea.

^*^Address correspondence to: kmpark@inu.ac.kr and dikim@skku.edu

^†^These authors contributed equally to this work.

**Table S1.** Abbreviations used in this paper.

| Abbreviation | Full name |
| --- | --- |
| ^1^H-NMR | Proton nuclear magnetic resonance |
| 2D | Two-dimensional |
| 3D | Three-dimensional |
| Apaf-1 | Apoptotic protease activating factor-1 |
| BAK | Bcl-2 homologous antagonist killer |
| BAX | Bcl-2-associated X |
| BCL-2 | B-cell lymphoma 2 |
| BCL-xL | B-cell lymphoma extra-large |
| BZ | Border zone |
| Ca(OH)_2_ | Calcium hydroxide |
| CaO_2_ | Calcium peroxide |
| CM | Conditioned medium |
| CO_2_ | Carbon dioxide |
| CYS | Cystamine dihydrochloride |
| D_2_O | Deuterium oxide |
| DIW | Deionized water |
| DMEM | Dulbecco’s modified Eagle’s medium |
| DPBS | Dulbecco's phosphate-buffered saline |
| DTT  ECM | DL-dithiothreitol  Extracellular matrix |
| EDC  EthD–1 | 1-Ethyl-3-(3-dimethylaminopropyl)carbodiimide hydrochloride  Ethidium homodimer-1 |
| EG-VEGF | Endocrine gland-derived vascular endothelial growth factor |
| FBS | Fetal bovine serum |
| FDA | Food and Drug Administration |
| FGF | Fibroblast growth factor |
| G′ | Elastic modulus |
| GAPDH | Glyceraldehyde 3-phosphate dehydrogenase |
| Gtn | Gelatin |
| GtnMI | Maleimide-conjugated gelatin |
| GtnSH | Thiolated gelatin |
| hADSC | Human adipose-derived stem cell |
| H&E | Hematoxylin and eosin |
| H_2_O_2_ | Hydrogen peroxide |
| HCl | Hydrochloric acid |
| HDF | Human dermal fibroblast |
| HGF | Hepatocyte growth factor |
| HIF-1α | Hypoxia-inducible factor 1-alpha |
| IACUC | Institutional Animal Care and Use Committee |
| IGFBP | Insulin-like growth factor-binding protein |
| IL-1β | Interleukin-1β |
| IZ | Infarcted zone |
| LAD | Left anterior descending coronary artery |
| LV | Left ventricle |
| LVEF | Left ventricular ejection fraction |
| LVFS | Left ventricular fractional shortening |
| LVIDd | Left ventricular internal dimension at end-diastole |
| LVIDs | Left ventricular internal dimension at end-systole |
| MHA | 6-maleimidohexanoic acid |
| MI | Myocardial infarction |
| MSC | Mesenchymal stem cell |
| NBCS | Newborn calf serum |
| NHS | N-hydroxysuccinimide |
| N.S | No significance |
| PBS | Phosphate-buffered saline |
| PCL | Polycaprolactone |
| PEG | Poly(ethylene glycol) |
| PO | Open/closed pocket patch-only group |
| P/S | Penicillin/streptomycin antibiotics |
| PU | Polyurethanes |
| qRT-PCR | Quantitative reverse transcription-polymerase chain reaction |
| RT | Room temperature |
| RZ | Remote zone |
| SD | Sprague-Dawley |
| SDS-PAGE | Sodium dodecyl sulfate-polyacrylamide gel electrophoresis |
| S.D. | Standard deviation |
| S_3DP | Stem cell spheroid-laden 3D patches |
| S_OP | Spheroid-laden open pocket patch |
| S_OPCL | Spheroid-laden open/closed pocket patch |
| SO | Sealant-only group |
| TCPS | Tissue culture polystyrene |
| TPU-CEC363 | Elastomeric bioscaffold |
| uPA | Urokinase-type plasminogen activator |
| UV  UTM | Ultraviolet  Universal testing machine |
| VEGF | Vascular endothelial growth factor |

**Table S2.** Sequences for primers used in qRT-PCR.

| **Species** | **Gene** | **Primer** | **Sequence (5’─3’)** |
| --- | --- | --- | --- |
| Human | GAPDH | Forward | GTC GGA GTC AAC GGA TTT GG |
|  |  | Reverse | GGG TGG AAT CAA TTG GAA CAT |
|  | BAX | Forward | GCT ACA GGG TTT CAT CCA GGA TC |
|  |  | Reverse | CCG TGT CCA CGT CAG CAA TC |
|  | BAK | Forward | CTC AGA GTT CCA GAC CAT GTT G |
|  |  | Reverse | CAT GCT GGT AGA CGT GTA GGG |
|  | Caspase-3 | Forward | CCT GGT TAT TAT TCT TGG CGA AA |
|  |  | Reverse | GCA CAA AGC GAC TGG ATG AA |
|  | Caspase-9 | Forward | CTC AGA CCA GAG ATT CGC AAA C |
|  |  | Reverse | GCA TTT CCC CTC AAA CTC TCA A |
|  | BCL-2 | Forward | TCC CTC GCT GCA CAA ATA CTC |
|  |  | Reverse | ACG ACC CGA TGG CCA TAG A |
|  | BCL-xL | Forward | AAC ATC CCA GCT TCA CAT AAC CCC |
|  |  | Reverse | GCG ACC CCA GTT TAC TCC ATC C |
|  | VEGF | Forward | GAG GGC AGA ATC ATC ACG AAG T |
|  |  | Reverse | CAC CAG GGT CTC GAT TGG AT |
|  | FGF-2 | Forward | AGC GGC TGT ACT GCA AAA AC |
|  |  | Reverse | GTA GCT TGA TGT GAG GGT CG |
|  | HGF | Forward | TCA AAT GCC AGC CTT GGA ATT CC |
|  |  | Reverse | TCA AGA GTG TAG CAC CAT GGC |
|  | HIF-1α | Forward | CAG TTA CGT TCC TTC GAT CAG TTG |
|  |  | Reverse | TTT GAG GAC TTG CGC TTT CA |

**
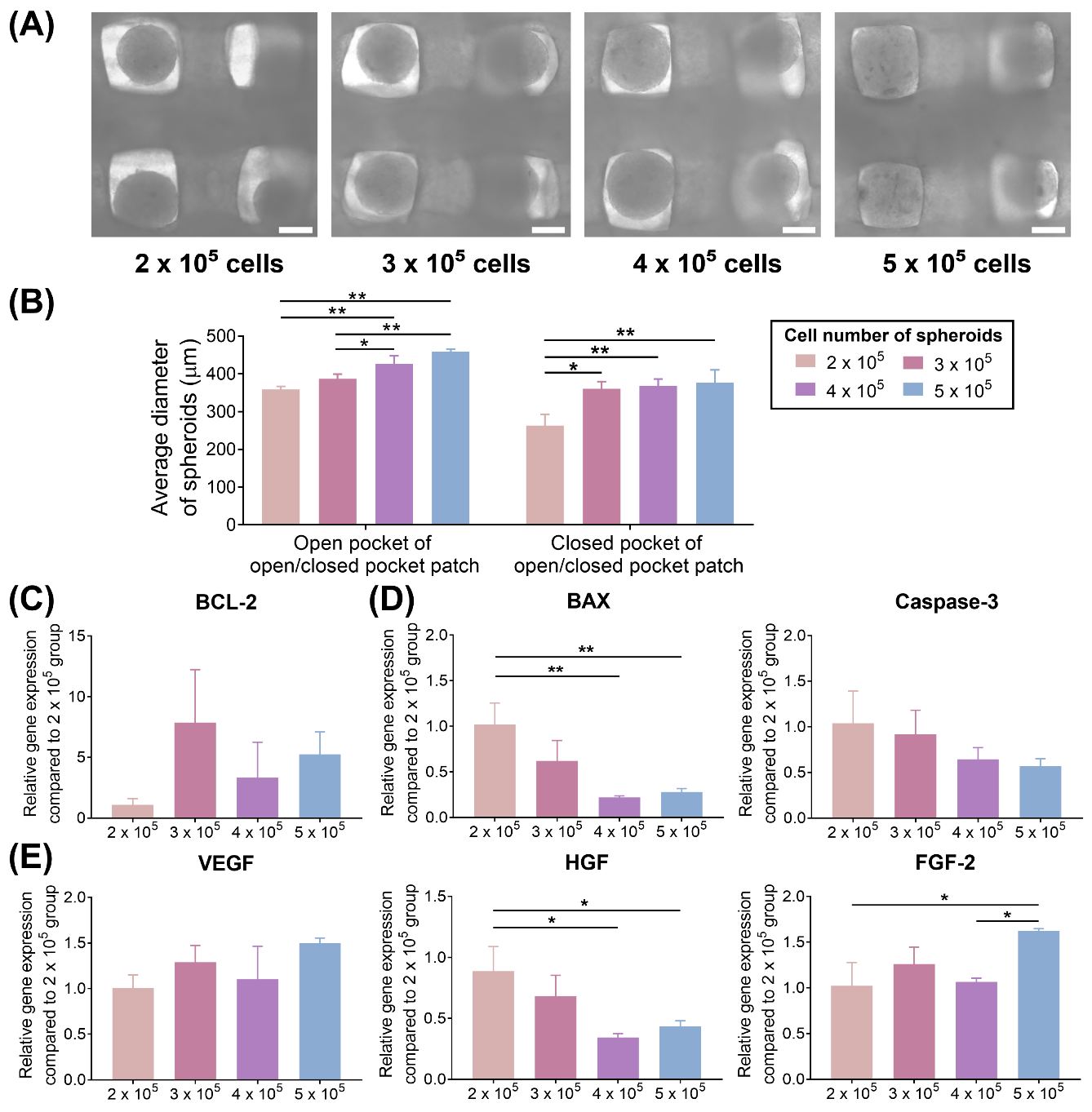
**

**Fig. S1. Optimization of the cell number to be loaded on the open/closed pocket patch.** (A) Representative optical images of spheroids according to cell number within open/closed pocket patch after 24 h. Scale bar: 200 μm. (B) The average diameter of spheroids formed according to the cell number in the open/closed pocket patch. The result in (B) is shown as the average values ± S.D. (n = 5). The gene expression of (C) anti-apoptotic factor (BCL-2), (D) apoptotic factors (BAX and Caspase-3), and (E) angiogenic growth factors (VEGF, HGF, and FGF-2) in spheroids formed according to the cell number. The results in (C), (D), and (E) are shown as the average values ± S.D. (n = 3). ∗ indicates a statistical significance compared to each group (*p < 0.05 and **p < 0.01).

**
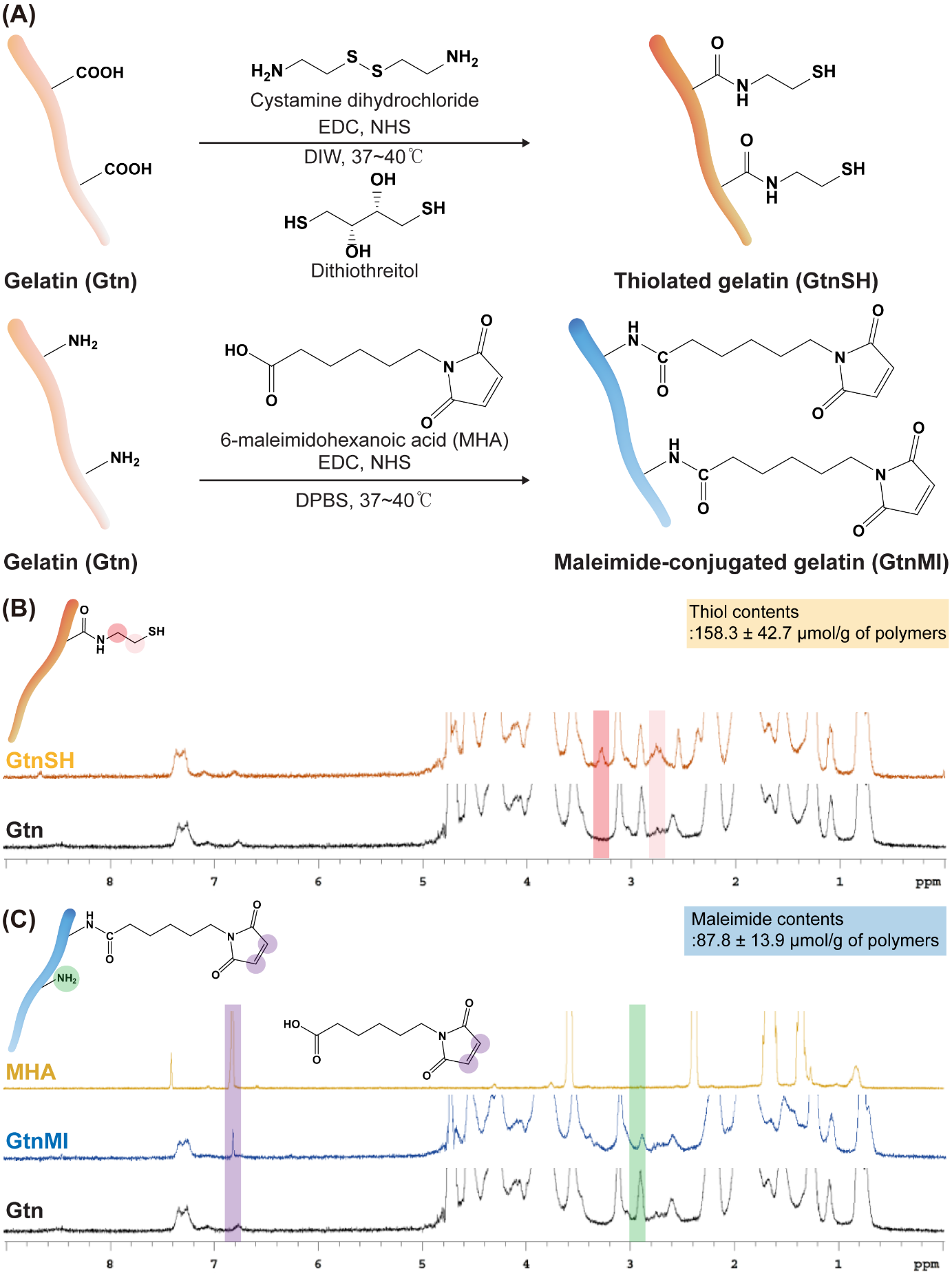
**

**Fig. S2. Synthesis and characterization of GtnSH and GtnMI.** (A) Schematic illustration of GtnSH and GtnMI synthesis using EDC/NHS chemistry. ^1^H NMR spectra of (B) GtnSH and (C) GtnMI with the content of each functional group.
